# A Collaborative Evaluation of LC-MS/MS Based Methods for BMAA Analysis: Soluble Bound BMAA Found to Be an Important Fraction

**DOI:** 10.3390/md14030045

**Published:** 2016-02-29

**Authors:** Elisabeth J. Faassen, Maria G. Antoniou, Wendy Beekman-Lukassen, Lucie Blahova, Ekaterina Chernova, Christophoros Christophoridis, Audrey Combes, Christine Edwards, Jutta Fastner, Joop Harmsen, Anastasia Hiskia, Leopold L. Ilag, Triantafyllos Kaloudis, Srdjan Lopicic, Miquel Lürling, Hanna Mazur-Marzec, Jussi Meriluoto, Cristina Porojan, Yehudit Viner-Mozzini, Nadezda Zguna

**Affiliations:** 1 wendy.beekman-lukassen@wur.nlmiquel.lurling@wur.nl; 2 maria.antoniou@cut.ac.cy; 3 blahova@recetox.muni.cz; 4 s3561389@ya.ru; 5 c.christoforidis@inn.demokritos.gra.hiskia@inn.demokritos.gr; 6 audrey.combes@espci.fr; 7 c.edwards@rgu.ac.uk; 8 jutta.fastner@uba.de; 9 joop.harmsen@wur.nl; 10 leopold.ilag@aces.su.senadezda.kiselova@aces.su.se; 11 kaloudis@eydap.gr; 12 slopicic@med.bg.ac.rs; 13; 14 biohm@ug.edu.pl; 15 jussi.meriluoto@abo.fi; 16 cristina.porojan@mycit.ie; 17 diti@ocean.org.il

**Keywords:** β-*N*-methylamino-l-alanine (BMAA), 6-aminoquinolyl-*N*-hydroxysuccinimidyl carbamate (AQC), α,γ-diaminobutyric acid (DAB), cycad, *Daphnia magna*, hydrophilic interaction liquid chromatography (HILIC), Internal standard, Liquid chromatography-tandem mass spectrometry (LC-MS/MS), *N*-(2-aminoethyl) glycine (AEG), phytoplankton, seafood

## Abstract

Exposure to β-*N*-methylamino-l-alanine (BMAA) might be linked to the incidence of amyotrophic lateral sclerosis, Alzheimer’s disease and Parkinson’s disease. Analytical chemistry plays a crucial role in determining human BMAA exposure and the associated health risk, but the performance of various analytical methods currently employed is rarely compared. A CYANOCOST initiated workshop was organized aimed at training scientists in BMAA analysis, creating mutual understanding and paving the way towards interlaboratory comparison exercises. During this workshop, we tested different methods (extraction followed by derivatization and liquid chromatography coupled to tandem mass spectrometry (LC-MS/MS) analysis, or directly followed by LC-MS/MS analysis) for trueness and intermediate precision. We adapted three workup methods for the underivatized analysis of animal, brain and cyanobacterial samples. Based on recovery of the internal standard D_3_BMAA, the underivatized methods were accurate (mean recovery 80%) and precise (mean relative standard deviation 10%), except for the cyanobacterium *Leptolyngbya*. However, total BMAA concentrations in the positive controls (cycad seeds) showed higher variation (relative standard deviation 21%–32%), implying that D_3_BMAA was not a good indicator for the release of BMAA from bound forms. Significant losses occurred during workup for the derivatized method, resulting in low recovery (<10%). Most BMAA was found in a trichloroacetic acid soluble, bound form and we recommend including this fraction during analysis.

## 1. Introduction

The neurotoxin β-*N*-methylamino-l-alanine (BMAA) is suspected to play a role in the progressive neurological diseases amyotrophic lateral sclerosis, Alzheimer’s disease and Parkinson’s disease [1,2,3,4]. Potential routes of human exposure to BMAA include contact with cyanobacteria infested surface waters and ingestion of BMAA containing food, such as fish and shellfish [5]. However, extensive research is needed to determine the precise role of BMAA in the etiology of these diseases along with characterization of pathways of human exposure.

To assess the health risk associated with BMAA, routes of human exposure are being quantified. BMAA can be present in natural phytoplankton (e.g., [6,7,8]) and can be taken up by aquatic organisms such as zooplankton [9,10,11], bivalves [12] and macrophytes [13]. Indeed, BMAA has been found in natural zooplankton and shellfish samples [7,14,15]. Moreover, it has been detected in other organisms from higher levels of the aquatic food web [7], including fish intended for human consumption [7,16]. Reported BMAA concentrations in phytoplankton and higher aquatic organisms vary widely, and a substantial part of this variation can be attributed to the use of nonselective analytical methods [17]. BMAA concentrations in aquatic organisms seem to lie within the ng/g dry weight (DW) to µg/g DW range in studies using well described analytical techniques supported by performance data [5].

Analytical procedures (method selectivity and sensitivity, fraction analyzed, quality control) play a critical role in assessing the putative link between BMAA and the abovementioned neurodegenerative diseases [18,19], as well as in the quantification of human exposure pathways [5]. Over the past years, many different analytical methods have been developed and at present, methods using tandem mass spectrometry (MS/MS) detection following proper sample processing are considered most suitable [5,17,20]. LC-MS/MS is currently the most frequently applied technique for BMAA analysis and within this technique, diverse sample processing and separation methods are used [5].

**Figure 1:**
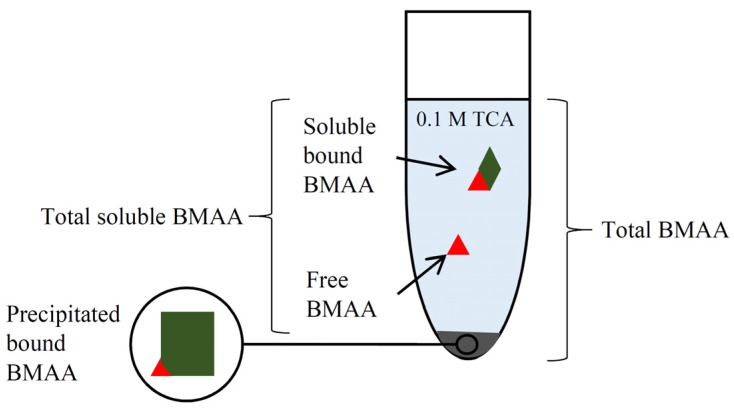
Terminology used in this manuscript for the different β-*N*-methylamino-l-alanine (BMAA) fractions. Free and soluble bound BMAA are found in the trichloroacetic acid (TCA) extract. Precipitated bound BMAA is found in the pellet created during extraction. Total BMAA is the sum of all fractions.

In natural samples, BMAA can be present as a free molecule or in bound forms. “Free BMAA” is the fraction obtained by extraction with polar solvents such as 0.1 M trichloroacetic acid (TCA) (Figure 1). Bound forms of BMAA can either stay in solution (“soluble bound BMAA”) or precipitate during extraction (“precipitated bound BMAA”) and BMAA can be released from both bound forms by acid hydrolysis (Figure 1). The total BMAA content of a sample is usually obtained by hydrolysis of the total sample (Figure 1). The precursor(s) of soluble bound BMAA have not been elucidated yet, but recently it was suggested that in mussels, soluble bound BMAA might not be bound to a peptide or protein [21]. The precursor(s) of the precipitated bound BMAA fraction are also unknown. This fraction is commonly referred to as “protein associated” or “protein bound” [22,23], but the association of BMAA with proteins in natural samples still needs to be elucidated. *In vitro*, BMAA can be incorporated into proteins and can be associated to proteins through non-covalent bonding [24,25], but *in vivo* experiments with bacteria do not show protein incorporation [26].

Only few studies look at soluble bound BMAA in an isolated fraction (e.g., [15,21,27,28]). In studies where total BMAA (*i.e.*, hydrolysis of the total sample) is (also) determined, ignoring soluble bound BMAA does not lead to an underestimation of the total BMAA concentration. However, when only free and precipitated bound BMAA are analyzed (e.g., [6,22,29] and more recently [11,12,30]) total BMAA concentrations might be underestimated, and the fate of BMAA in experimental systems might be difficult to assess. As an example, in a recent study on BMAA metabolism in the macrophyte *Ceratophyllum demersum*, in which only free and precipitated bound BMAA were analyzed, detectable BMAA concentrations in the exposed plants dropped during depuration, while no BMAA was found in the depuration medium and BMAA catabolism did not seem to have occurred. This lead the authors to conclude that BMAA was likely covalently bound in a form undetectable by the analytical methods employed [30].

After extraction, BMAA can be analyzed by LC-MS/MS without derivatization. As BMAA is a small, polar molecule, hydrophilic interaction liquid chromatography (HILIC) is in these cases predominantly used for separation (e.g., [6,15,31,32,33]). BMAA can also be derivatized after extraction to obtain a larger, more hydrophobic molecule which is easily separated by reversed phase liquid chromatography. Commonly used derivatization agents are 6-aminoquinolyl-*N*-hydroxysuccinimidyl carbamate (AQC, e.g., [8,34,35]), propyl chloroformate (e.g., [26,36]) and dansyl chloride [14,37].

As outlined above, analytical chemistry plays an essential role in BMAA risk assessment, but to date, method harmonization and inter-laboratory comparison of methods have not yet been performed. During a workshop organized in Wageningen University under the auspices of the CYANOCOST network (COST Action ES 1105), analysts from different labs were trained in BMAA analysis and BMAA methods were discussed. By doing so, we aimed to create mutual understanding and to pave the way towards an inter-laboratory comparison exercise and ultimately towards method harmonization. During this workshop, samples from four relevant matrices (cycad, animal, brain and cyanobacteria) were extracted with at least two different methods (one followed by derivatization before LC-MS/MS analysis and one directly followed by LC-MS/MS analysis), and each workup was performed by two pairs of analysts. All samples were analyzed by LC-MS/MS by one operator. The analysts were experienced in cyanotoxin analysis, were provided with detailed protocols and instructions and were intensively supported by the three trainers who had developed the methods used.

## 2. Experimental Design

Three different sample types, animal samples (seafood and BMAA exposed *Daphnia magna*), brain tissue (unspiked and spiked with BMAA before workup) and cyanobacterial samples (*Leptolyngbya* PCC 73110 and an *Anabaena* dominated field sample), were prepared for underivatized and AQC derivatized LC-MS/MS analysis (detailed Materials and Methods are described in Supplementary Material S1 (underivatized protocols) and Supplementary Material S2 (derivatized protocol)). We selected sample preparation methods that were published, validated and developed by the trainers of the workshop (see [17] for underivatized analysis of animal and cyanobacterial samples, [38] for underivatized analysis in brain and [16] for AQC derivatized analysis of all sample types). Where needed, the extraction methods were adapted to the available equipment.

The sample preparations were performed by the workshop participants. An open call was distributed through the CYANOCOST network and the selection of participants was carried out jointly by CYANOCOST Working Group 3: “Cyanotoxin analysis” leaders and by the local organizers. Selection was largely based on the applicants’ experience with cyanotoxin analysis, and especially with LC-MS/MS analysis. During the workshop, the following measures were taken to minimize any variation caused by lack of training: Before starting the practical work, all participants attended lectures on the chemical properties of BMAA and on methods of BMAA analysis. All participants were given detailed documented protocols for the different extraction methods and were trained in the techniques and instrumentation used. Constant technical support was provided by three trainers who developed the sample preparation (Ilag/Zguna for protocol D, Combes for protocol B and Faassen for protocol A and C) and by laboratory technicians who had experience with the methods used. All LC-MS/MS analyses were performed on an Agilent 1260 LC coupled to an Agilent 6460 triple quadrupole mass spectrometer by one operator.

**Figure 2:**
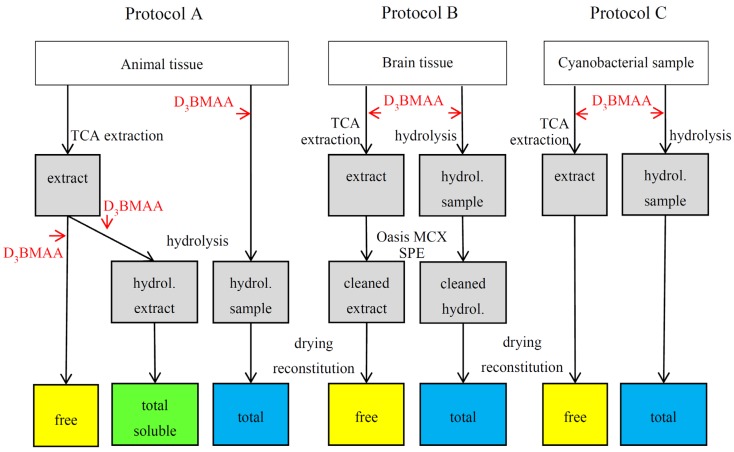
Sample preparation schemes for the analysis of underivatized BMAA in three different matrices: animal tissue other than brain (protocol A), brain tissue (protocol B) and cyanobacterial samples (protocol C). The workup for total BMAA is the same in method A and C. Workup for free BMAA in these protocols only differs in the point at which D_3_BMAA was added.

The samples that were prepared for underivatized LC-MS/MS analysis were extracted with 0.1 M TCA at ambient temperature to obtain free BMAA. Total BMAA was obtained by 6 M HCl hydrolysis of the total sample. For the animal samples, total soluble BMAA was also determined by hydrolyzing the dried 0.1 M TCA extract with 6 M HCl. This fraction was not determined for the other two sample types because we did not have brain and cyanobacterial samples with relatively high BMAA concentrations. The workup for the brain samples included an additional cleanup step by Oasis MCX solid phase extraction (SPE, Figure 2).

In all protocols, D_3_BMAA was added as internal standard, and blanks (workup without matrix, negative controls) and cycad seed sarcotesta (positive controls) were included. All samples and controls were prepared in triplicate by two pairs of analyst, resulting in six workups per sample (see Table S1.1 in Supplementary Material S1).

We intended to use the derivatized protocol for total BMAA determination in all sample types. However, in agreement with a recent method evaluation in an independent laboratory [39], we obtained such a poor recovery with the derivatized protocol (Protocol D, recovery < 10%) that we did not use it for BMAA quantification. From this point on, the manuscript therefore focuses on the underivatized protocols, and the results and discussion for the derivatized protocol can be found in Supplementary Material S2.

## 3. Results and Discussion

### 3.1. Trueness and Precision

Trueness of protocols A, B and C, expressed as mean recovery of D_3_BMAA added before workup, were not all within the acceptable range of 70%–120% [40] (Table 1). Some fractions of the control samples gave a slightly lower recovery (between 59% and 69%) and D_3_BMAA recovery in *Leptolyngbya* was very low (7%–21%). Better recoveries (88%–100% for the free fraction and 56%–75% for the total samples) had previously been obtained for cyanobacterial labstrains extracted with the same protocol [17] and it is unclear what has caused the low recovery in this *Leptolyngbya* strain. In contrast to *Leptolyngbya*, D_3_BMAA recovery from the free fraction in *Daphnia* (141%) was too high. When the workup was repeated, D_3_BMAA recovery was well within the acceptable range (103%, SD 7.4, *n* = 3).

Intermediate precision (within-laboratory reproducibility, expressed as relative standard deviation of D_3_BMAA recovery) was below 10% for most, and below 20% for all samples except for *Leptolyngbya* (Table 1). The workup in protocol A and C was essentially the same for free BMAA and exactly the same for total BMAA, but the extractions were performed on different days. When the results of protocols A and C were combined, the precision was still within the acceptable range: 9.8% for D_3_BMAA recovery in the free fraction in blanks, 9.4% in the free fraction of cycads, 19.5% in the total fraction in blanks (all *n* = 12) and 15.1% in the total fraction of cycads (*n* = 11).

In Table 1, trueness and intermediate precision were based on the recovery of D_3_BMAA that was added as a free compound, as no “bound” D_3_BMAA or BMAA is available. When intermediate precision is expressed as the relative standard deviation of the amount of BMAA found in the positive control (cycad seed), which does contain bound forms of BMAA, it shows that in all three protocols, intermediate precision for total BMAA is greater than 20% and that correction for D_3_BMAA recovery does not increase precision (Table 2). For total BMAA determination, D_3_BMAA recovery and the BMAA concentrations uncorrected for D_3_BMAA recovery were not correlated (Pearson product moment correlation, *p* = 0.15, *n* = 17, see Supplementary Figure S3), in contrast to the free fraction, where this correlation did exist (correlation coefficient 0.88, *p* < 0.001, *n* = 18, see Supplementary Figure S3). Assuming that the stability of (free) BMAA and D_3_BMAA is the same, this implies that during workup for total BMAA (and possibly also for soluble bound BMAA), small procedural variations have affected the release or formation of BMAA, but not, or to a lesser extent, its stability or signal suppression. This also suggests, that although each method seemed precise and accurate based on D_3_BMAA recovery, correction for D_3_BMAA recovery only results in accurate quantification of free BMAA and not in accurate quantification of bound forms. (Free) D_3_BMAA added before sample procession does therefore seem to be a good indicator for losses during extraction and changes in MS/MS signal due to matrix effects, but does not seem to accurately reflect the release or formation of bound BMAA in natural samples.

### 3.2. BMAA in Blanks and Cycad Samples

No BMAA was detected in any of the blanks (negative controls). BMAA was detected in the cycad seed (positive controls), free BMAA concentrations averaged 8.8 µg/g DW (SD 1.8, *n* = 18), which is similar to the value previously determined in the same sample (10.7 µg/g DW, SD 2.9, *n* = 3 [17]).

**Figure 3:**
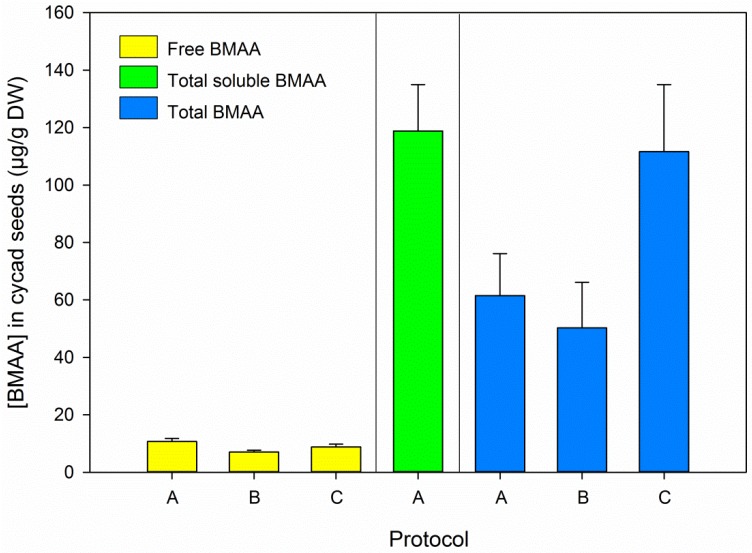
BMAA concentrations in cycad seeds as determined by protocols A to C, results for both pairs are combined. Error bars represent standard deviations, *n* = 6, except for total BMAA determined by protocol A, where *n* = 5. “Total soluble BMAA” refers to the TCA soluble fraction, including free BMAA.

BMAA was found in the hydrolyzed 0.1 M TCA extract (“total soluble BMAA” in Figure 3), and total soluble BMAA exceeded the total BMAA concentration (*t*-test total soluble *vs.* total BMAA, *t*_21_ = 3.071, *p* = 0.006, *n* = 23, Figure 3). Although the average total BMAA concentration in the cycad seed as determined by all three protocols (75.2 µg/g DW, SD 33.1, *n* = 17) was consistent with previously reported values for this sample (75.0 µg/g DW, SD 10.8, *n* = 3, [17]), these values differed substantially between the protocols used in this study (Figure 3). This implies that the release of BMAA from precursor bound forms, for which the addition of free D_3_BMAA as an internal standard does not correct, is sensitive to slight variations in the workup procedure. In our study, hydrolysis was performed overnight and incubation times were not strictly controlled or registered. Although different hydrolysis procedures are currently applied by different labs [20], the effects of variations in hydrolysis conditions have not been systematically evaluated yet. Given the variation observed in the total BMAA determinations our study, this might be worth looking into. This work should be carried out with samples containing bound forms of BMAA, preferably matrix matched certified reference materials. Such materials are not available yet, but the recent finding of BMAA in commercially available mussel material [41] is promising. Until certified reference materials are available, samples that contain a relatively high concentration of bound BMAA, such as cycad seeds, can be used during method development and comparison.

### 3.3. BMAA in Brain Tissue

No BMAA was detected in the unspiked brain samples. An additional set of brain samples was therefore spiked with BMAA before sample preparation. After TCA extraction, a BMAA concentration of 3.0 µg/g DW (SD 0.1, *n* = 6) was determined, which was exactly the spiked concentration. The BMAA concentration determined after hydrolysis of the total sample was 39.9 µg/g DW (SD 3.1, *n* = 6), which is very close to the spiked concentration of 40 µg/g DW. These findings support our assumption (see Section 3.1) that BMAA and D_3_BMAA added before workup (*i.e.*, the free compounds) behave similar in terms of stability and signal suppression, both during 0.1 N TCA extraction and during 6 M HCl hydrolysis.

### 3.4. BMAA in Animal and Cyanobacterial Samples

No BMAA was detected in any of the cyanobacterial samples. The *Leptolyngbya* strain used in this study had been shown to contain BMAA at concentrations below 1 µg/g DW with AQC derivatized LC-MS/MS methods [35,39], but no BMAA was detected in the same strain by underivatized LC-MS/MS analysis ([15], LOD 0.225 µg/g DW). We did not detect BMAA in this strain, but this might be attributed to the high LOD for this sample (estimated at 1 µg/g DW for free BMAA and 20 µg/g DW for total BMAA, as opposed to 0.2 µg/g DW for free BMAA and 2.5 µg/g DW for total BMAA in *Anabaena* field samples), which was caused by low recovery in *Leptolyngbya*.

**Figure 4:**
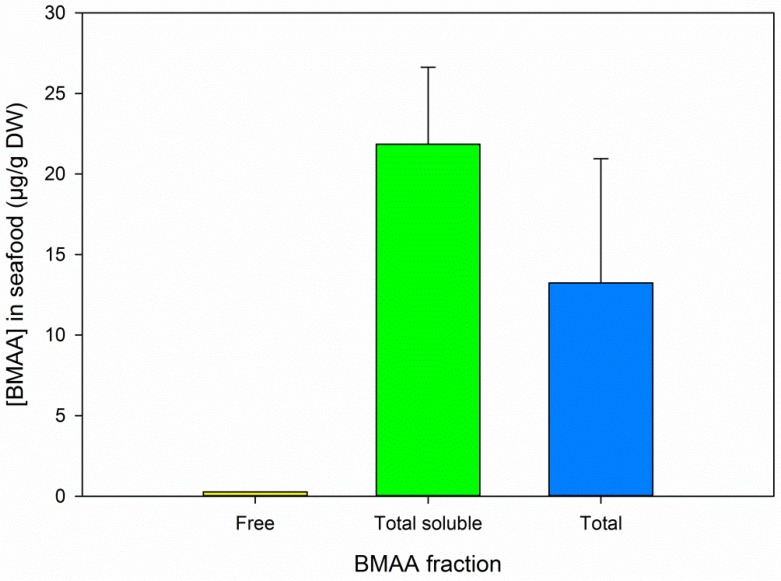
BMAA concentrations in seafood samples as determined by protocol A, results for both pairs are combined. Error bars represent standard deviations, *n* = 1 for free BMAA and *n* = 6 for each of the other two fractions. “Total soluble” refers to the TCA soluble fraction, including free BMAA.

In seafood samples, free BMAA was detected in two replicates, of which one was quantifiable at a concentration of 0.3 µg/g DW. Highest BMAA concentrations were again found in the hydrolyzed TCA extract (*t*-test total soluble *vs.* total BMAA, *t*_10_ = 2.330, *p* = 0.042, *n* = 12, Figure 4). The variation within each fraction was considerable: relative SD of 21.8 for soluble bound BMAA and 58.2 for total BMAA, where the relative SD of D_3_BMAA recovery was below 8% for both fractions (Table 1). It is most likely that this variation is caused by small variations during workup (as discussed in Section 3.1 and Section 3.2) and possibly by sample heterogeneity, for both of which the addition of an internal standard cannot correct.

**Figure 5:**
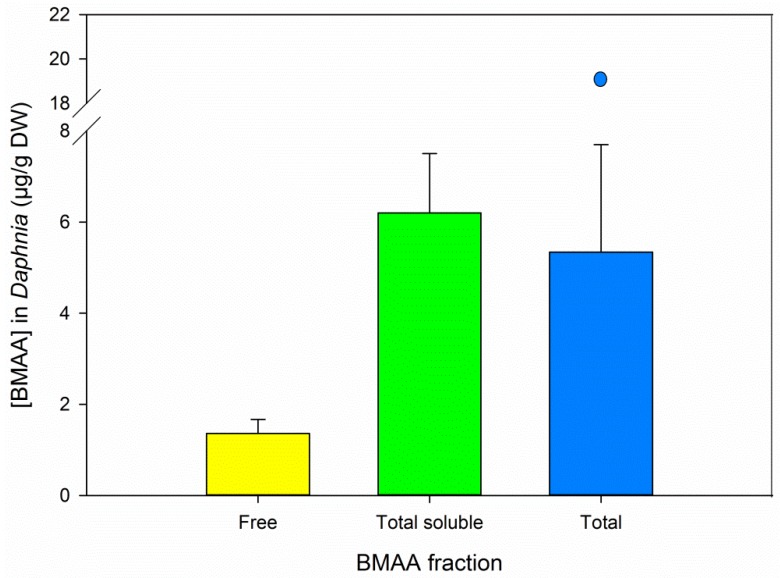
BMAA concentrations in BMAA exposed *Daphnia* as determined by protocol A, results for both pairs are combined. Error bars represent standard deviations, *n* = 6, except for total BMAA, where *n* = 5 and the sixth data point is presented as outlier. “Total soluble” refers to the TCA soluble fraction, including free BMAA.

All *Daphnia* samples contained quantifiable amounts of free and total soluble BMAA (Figure 5). Total soluble BMAA concentrations equaled total BMAA concentrations (with outlier included: Mann–Whitney rank sum test, *U* = 15, *p* = 0.699, *n* = 12; without outlier: *t*-test total soluble *vs.* total BMAA, *t*_9_ = 0.768, *p* = 0.462, *n* = 11, Figure 5). The variation observed in the total BMAA results may be due to sample heterogeneity along with differences in actual sample size (tissue weight) due to incomplete drying of the animals. Unexposed *Daphnia* and their food source *Scenedesmus obliquus* did not contain detectable amounts of BMAA [9,17].

### 3.5. BMAA Fractions

Free BMAA was found in all cycad and *Daphnia* samples, and in two of the six seafood replicates. Although free BMAA can slowly be released from bound forms during extraction with dilute acid at low temperatures [21], we do not expect that this process has substantially added to the free BMAA concentration we found as our handling times during TCA extraction were short (less than one hour).

In the BMAA positive samples we analyzed, total soluble BMAA concentrations (free and soluble bound BMAA, represented by the green bars in Figure 3, Figure 4 and Figure 5) were relatively close to the total BMAA concentrations (blue bars in same figures). The tested samples are therefore not expected to contain a high percentage of precipitated bound BMAA. However, a direct comparative analysis of free, soluble bound and precipitated bound BMAA is needed to definitively answer this question.

The form in which soluble bound BMAA was present in the hydrolyzed extract is unclear, because from our experiment we can only derive that it was TCA soluble and that it was bound to a precursor. Whether it is the same low molecular weight, non-protein/peptide precursor as found in mussels [21] is unknown. Further work is needed to identify the structure(s) of this precursor, and to optimize its extraction, as milder methods than the 6 M HCl liquid hydrolysis used in this study have been shown to release soluble bound BMAA in mussels [21].

We detected soluble bound BMAA in all three BMAA positive samples (cycad, seafood and exposed *Daphnia*). Although a limited number of studies have determined this fraction so far, soluble bound BMAA seems to occur in a diversity of organisms: cycad seeds (this study and [27]), periphyton [28], plankton [8,28], and bivalves [15,21,28]. It is therefore recommended to include soluble bound BMAA in future studies, for instance by hydrolyzing the total sample (e.g., [14,16,17]), or by releasing it from the dried extract [8,15,21,28]. When only free and precipitated bound BMAA are determined, the soluble bound fraction can be overlooked, potentially resulting in a substantial underestimation of the total sample’s BMAA content.

## 4. Conclusions and Outlook

The three LC-MS/MS based protocols we tested to analyze underivatized BMAA in animal tissue, brain tissue and cyanobacterial samples were generally accurate and precise in terms of D_3_BMAA recovery, as well as for free BMAA determination in the positive control (cycad seeds). However, total BMAA determination in cycad seeds was less precise (intermediate precision ranging from 20% to 32%). We suspect that small variations during workup have influenced the liberation or formation of BMAA from bound forms, for which the addition of free D_3_BMAA as internal standard could not correct. Given the observed variation in total BMAA concentrations in cycad seeds, we recommend optimization of the workup for soluble bound and total BMAA, which should be performed with samples containing bound BMAA.

The majority of the BMAA detected in the positive samples (cycad seeds, seafood and *Daphnia*) was present in a bound form in the TCA extract. This fraction was released by liquid phase acid hydrolysis, but additional work is needed to identify the structure of its precursor(s) and to optimize its extraction. When only free and precipitated bound BMAA are determined, this fraction will be overlooked. Until its structure has been elucidated and extraction has been optimized, we recommend to include soluble bound BMAA either by determining total BMAA or by hydrolyzing (part of) the extract used for free BMAA analysis.

During the workshop, scientists from 12 different research groups were provided with the knowledge and skills to develop appropriate BMAA methods in their own laboratories. Furthermore, mutual understanding was created by an open discussion on the pros and cons of different analytical techniques and by evaluation of the conflicting data in BMAA literature. This common starting point will facilitate the performance of interlaboratory comparison exercises, which are needed to progress BMAA research [5]. 

## Figures and Tables

**Table 1:** Trueness (mean D_3_BMAA recovery (%)) and intermediate precision (relative standard deviation of D_3_BMAA recovery, *n* = 6, results of both pairs combined), for samples prepared for underivatized analysis. Trueness outside the acceptable range is indicated with blue (<70%) and red (>120%). Precision exceeding the acceptable value (20) is indicated with red [40].

Protocol	Animal (A)			Brain (B)		Cyanobacteria (C)	
Fraction	Free	T.S. ^1^	Total	Free	Total	Free	Total
Blank	85 (2.6)	65 (4.9)	81 (13.7)	78 (4.8)	72 (8.4)	100 (7.8)	59 (6.3)
Cycad	93 (7.8)	64 (11.4)	86 (2.1) *	69 (7.5)	73 (2.5)	103 (8.5)	65 (4.3)
Seafood	96 (6.6)	78 (7.9)	108 (6.7)	-	-	-	-
*Daphnia magna*	141 (2.5)	75 (1.0)	110 (8.0)	-	-	-	-
Brain unspiked	-	-	-	77 (11.1)	84 (15.7)	-	-
Brain spiked	-	-	-	80 (6.0)	82 (9.0)	-	-
*Anabaena*	-	-	-	-	-	103 (7.4)	78 (2.3)
*Leptolyngbya*	-	-	-	-	-	21 (61.0)	7 (41.5)

^1^ Total Soluble, * *n* = 5.

**Table 2:** Intermediate precision expressed as relative standard deviation of the BMAA concentration (µg/g DW) determined in cycad seed by underivatized analysis, data with and without correction for D_3_BMAA recovery are shown (*n* = 6, results of both pairs combined). Results exceeding the acceptable value (20, [40]) are indicated with red.

Protocol	Animal (A)			Brain (B)		Cyanobacteria (C)	
Fraction	Free	T. S. ^1^	Total	Free	Total	Free	Total
uncorrected for D_3_BMAA	10.3	8.4	22.9 *	13.5	31.4	18.5	20.5
corrected for D_3_BMAA	10.4	13.6	23.9 *	9.2	31.6	11.6	20.9

^1^ Total Soluble, * *n* = 5.

## Supplementary Files

Supplementary File 1
