# Supplementary material for: A Collaborative Evaluation of LC-MS/MS Based Methods for BMAA Analysis: Soluble Bound BMAA Found to Be an Important Fraction
Source: Mar Drugs. 2016 Feb 29;14(3):45. doi: 10.3390/md14030045 (PMC4820299; doi:10.3390/md14030045)
Supplement: Supplementary File 1 [file marinedrugs-14-00045-s001.pdf]

marine drugs

Supplementary Materials: A Collaborative Evaluation of LC-MS/MS Based Methods for BMAA Analysis: Soluble Bound BMAA Found to Be an Important Fraction

Elisabeth J. Faassen, Maria G. Antoniou, Wendy Beekman-Lukassen, Lucie Blahova, Ekaterina Chernova, Christophoros Christophoridis, Audrey Combes, Christine Edwards, Jutta Fastner, Joop Harmsen, Anastasia Hiskia, Leopold L. Ilag, Triantafyllos Kaloudis, Srdjan Lopicic, Miquel Lürling, Hanna Mazur-Marzec, Jussi Meriluoto, Cristina Porojan, Yehudit Viner-Mozzini and Nadezda Zguna

S1: Detailed methods underivatized protocols

S.1.1. Experimental Design

Table S1.1. Experimental design. Extraction for underivatized LC-MS/MS analysis was carried out by two pairs of analysts (1 to 6) for animal tissue (protocol A), brain tissue (B) and cyanobacterial samples (C). Extraction for derivatized LC-MS/MS analysis was similar for all sample types (D) and was also carried out by two pairs of analysts.

**Protocol**	**A**	**A**	**A**	**B**	**B**	**C**	**C**	**D**
BMAA fraction	free	t.s. [1]	total	free	total	free	total	total
Blank (neg. control)	5,6	5,6	1,2	1,2	3,4	3,4	5,6	1,3
Cycad seed (pos. control)	5,6	5,6	1,2	1,2	3,4	3,4	5,6	2,4
Seafood	5,6	5,6	1,2	-	-	-	-	3,4
Daphnia magna	5,6	5,6	1,2	-	-	-	-	3,4
Brain unspiked	-	-	-	1,2	3,4	-	-	5,6
Brain spiked	-	-	-	1,2	3,4	-	-	5,6
Anabaena	-	-	-	-	-	3,4	5,6	1,2
Leptolyngbya	-	-	-	-	-	3,4	5,6	1,2

[1] t.s.: total soluble BMAA.

S.1.2. Sample origin and storage

The positive control sample consisted of the sarcotesta of *Cycas micronesica* (Hill) seed, which was kindly provided by Chad Husby, Montgomery Botanical Centre, Miami, US. The seed was freeze dried, homogenized by mortar and pestle and stored at −20 °C. The seafood sample was a mixture of crabmeat and Asari clam (*Venerupis philippinarum*), kindly provided by Stephanie Christensen, University of Hawaii, US. The samples were mixed to obtain enough biomass for all analyses. Crabmeat was purchased at a seafood market in Louisiana (US) and shipped to Hawaii frozen on dry ice. It was stored at −80 °C until freeze drying. The freeze dried sample was hand ground with mortar and pestle, and stored in the dark at room temperature. The Asari clam was purchased from a seafood market at Nijiya market, Hawaii, US and prepared and stored the same way as the crabmeat. *Daphnia magna* (Strauss) was isolated from the Dutch lake Zwemlust in 1999, it was maintained in jars containing RT medium [1] and fed with the green algae *Scenedesmus obliquus* SAG 276/3a. Prior to the experiment, the animals were kept under similar conditions, but BMAA (L-BMAA hydrochloride, Sigma) was added to the jars. The animals were exposed to a nominal concentration of 78 μg/L for approximately two weeks. After exposure, animals were rinsed with water, freeze dried and homogenized with a plastic stick. The brain sample was kindly provided by Lonneke IJsseldijk, Utrecht University, The Netherlands and by Mardik Leopold, IMARES, The Netherlands. It was

taken from a stranded male harbour porpoise (*Phocoena phocoena,* 33.3 kg, 134 cm), found in Callantsoog (The Netherlands) on 16 June 2008. The animal was still relative fresh (DCC 2), had slightly lost weight (NCC 3) and had probably died of pneumonia. The corpse was kept at −20 °C until dissection and the whole brain was then stored at the same temperature. Before the start of the experiment, the brain was freeze dried and homogenized in a food processor. The *Anabaena* dominated scum sample was collected from a Dutch lake in 2008 and was stored at −20 °C after freeze drying. The lab strain *Leptolyngbya* PCC 73110 was kindly provided by Birgitta Bergman, Stockholm University, Sweden and was grown at 20 °C on BG11 growth medium [2]. After collection by centrifugation and freeze drying, the samples were stored at −20 °C. Samples were prepared and analyzed as described in the sections below.

S.1.3. Protocol A

The protocol used for extraction of animal samples followed by underivatized LC-MS/MS analysis was adapted from a method developed and validated for the underivatized analysis of cyanobacterial samples [3]. Main adaptations are that the total soluble BMAA fraction was included, and that analysis was performed on a more recent LC-MS/MS system, with enhanced sensitivity.

For the extraction of free BMAA and the TCA soluble fraction released by hydrolysis (the "total soluble fraction"), 8 mg of cycad and 10 mg of *Daphnia* and seafood was used. Samples were extracted by addition of 600 μL 0.1 M TCA, vortexed and left for 10 min at room temperature. Following a further vortex, samples were centrifuged for 5 min at 16000× *g* and the supernatant was transferred to an Eppendorf tube with a 0.2 μm cellulose acetate filter (Grace Davison Discovery Science, Columbia, USA) and centrifuged for 5 min at 16000× *g*. TCA (600 μL) was then added to the pellet, and after vortexing, centrifugation and filtration as described above, both extracts were combined. For the analysis of free BMAA, 20 μL of a 2 mg/L D_3BMAA (D_3BMAA hydrochloride, Novakits, Nantes, France) solution was added to 600 μL of the pooled extract. The extract was subsequently dried in a speedvac (SPD121P, Thermo Scientific Savant, Asheville, USA) and reconstituted in 500 μL water/acetonitrile/formic acid (*v*/*v* 33:67:0.1).

For the analysis of the total soluble fraction, 120 μL of the pooled extract was transferred to a small glass tube, and 40 μL of the 2 mg/L D_3BMAA solution was added. This extract was freeze-dried, and 30 μL 6 M HCl was added to the dry sample. After flushing the sample with nitrogen, it was hydrolyzed overnight under vacuum at 105 °C in a hydrolysis workstation (Eldex). After hydrolysis, the samples were dried under vacuum, dissolved in 1000 μL water/acetonitrile/formic acid (*v*/*v* 33:67:0.1) and filtrated over a 0.2 μm cellulose acetate filter.

Total BMAA was determined in 0.8 mg of cycad seeds and 1 mg of the *Daphnia* and seafood samples. An aliquot of the same D_3BMAA solution (40 μL) was added and the sample was dried under vacuum. Samples were hydrolyzed by addition of 6 M HCl (30 μL) and reconstituted as described above for the total soluble fraction.

S.1.4. Protocol B

The protocol used for brain samples followed by underivatized LC-MS/MS analysis was adapted from a method developed and validated for the underivatized analysis of free BMAA in brain tissue [4]. Main adaptations to this published method are that we included a method for total BMAA, that we started with freeze dried samples instead of with samples that were only frozen and thawed and that we therefore adapted the first extraction steps for free BMAA, and that the LC-MS/MS analysis was performed according to [5] on the same LC-MS/MS system as used for the other analyses in this experiment.

Free BMAA was determined in 8 mg of cycad seed, and 20 mg of harbour porpoise brain. Each pair analyzed three unspiked brain samples, three other samples were spiked with 60 ng L-BMAA directly after weighing. An aliquot (40 μL) of the 2 mg/L D_3BMAA solution and 3 mL 0.1 M TCA were added, and the samples were vortexed. BMAA was extracted in an ultrasonic bath (Branson 3510, Danbury, USA) at room temperature for 10 min, after which the sample tubes were centrifuged for 10 min at 3500× g. The supernatant was transferred to a clean glass tube, and solid phase extraction

(SPE) was performed using MCX, 60 mg, 3 mL cartridges (Oasis, Etten-Leur, The Netherlands). Cartridges were conditioned with 2 mL of methanol, followed by 1 mL of water with formic acid (pH = 3). Sample (3 mL) was then loaded onto the cartridges, which were subsequently washed with 1 mL cyclohexane. After drying the cartridges with nitrogen gas, 1 mL 0.1 M HCl and 2 mL methanol were added. The samples were then eluted with 3 mL methanol with NH_4OH (freshly prepared by adding 6.6% of a 25% NH_4OH solution to 93.4% of methanol (*v*/*v*)). After drying in the speedvac, the samples were reconstituted in 1000 µL of water/acetonitrile/formic acid and filtered as described above.

Total BMAA was determined in 0.8 mg of cycad seeds and in 1 mg of brain samples. Directly after weighing, three replicate brain samples for each pair were spiked with 40 ng L-BMAA. An aliquot (40 µL) of the 2 mg/L D_3BMAA solution was added to all samples, and after drying, hydrolysis was performed as described in Section S.1.3. After hydrolysis, dried samples were quantitatively transferred to new tubes using 0.1 M TCA, final volume was 3 mL. Samples were subsequently cleaned up by SPE and reconstituted as described above.

S.1.5. Protocol C

The protocol used for cyanobacterial samples followed by underivatized LC-MS/MS analysis was described and validated previously [3], but for this experiment, a more updated LC-MS/MS system was used [5].

Free BMAA was determined in 4 mg of cycad seeds and 5 mg of cyanobacterial samples. To each sample, 20 µL of a 2 mg/L D_3BMAA solution was added. Samples were extracted as described for the animal samples, but only 300 µL of 0.1 M TCA was used during both extraction steps, instead of 600 µL. After extraction and filtration, the complete extract was dried in a speedvac, and reconstituted in 500 µL water/acetonitrile/formic acid (*v*/*v* 33:67:0.1).

Total BMAA determination was the same as described for the animal samples (method A, Section S.1.3), the amount of cycad samples used was 0.8 mg, and 1 mg was used for the cyanobacterial samples.

S.1.6. LC-MS/MS Analysis

LC-MS/MS analyses were performed on an Agilent 1260 LC coupled to an Agilent 6460 triple quadrupole mass spectrometer. The method for underivatized analysis is described in [5]. Separation was performed with a 2.1 × 150 mm, 5 µm ZIC-HILIC column (SeQuant, Solna, Sweden). Column temperature was 40 °C, injection volume 5 µL and flowrate 0.4 mL/min. The mobile phase consisted of acetonitrile with 0.1 % formic acid (*v*/*v*, eluent A) and water with 0.1% formic acid (*v*/*v*, eluent B). The elution program was 0–2 min: 95% A, 4 min: 65% A, 8–17 min 55% A, 17–23 min 95% A, with linear decreases between the steps. During the first 4 and last 6 minutes the flow was directed to waste. Nitrogen was used as drying, sheath and collision gas and source settings were: drying gas temperature 230 °C, drying gas flow 12 L/min, nebulizer pressure 40 psi, sheath gas temperature 250 °C, sheath gas flow 12 L/min, capillary voltage 2500 V, nozzle voltage 500 V. Both quadrupoles were operated in unit mode and the ESI source was operated in positive mode. MS/MS settings, and precursor to product ion transitions monitored in multiple reaction monitoring (MRM) for BMAA, D_3BMAA, α,γ-diaminobutyric acid (DAB, DAB dihydrochloride, Sigma, Zwijndrecht, The Netherlands) and *N*-(2-aminoethyl) glycine (AEG, TCI) are shown in Table S1.2.

Table S1.2. MS/MS settings and MRM transitions for underivatized analysis.

Compound	Precursor	F [1]	Quant [2]	CE [3]	Qual [4]	CE	Ratio [5]	Qual	CE	Ratio
	m/*z*	V	*m*/*z*	V	*m*/*z*	V	%	*m*/*z*	V	%
D_3BMAA	122.1	50	105.1	4	88.1	8	27	76.2	8	43
BMAA	119.1	50	102.1	4	88.1	8	25	76.2	8	25
DAB	119.1	50	101.1	4	74.2	4	-	-	-	-
AEG	119.1	50	102.1	4	-	-	-	-	-	-

[1] Fragmentor voltage, [2] Quantifier ion, [3] Collision energy, [4] Qualifier ion, [5] Ratio between areas of qualifier and quantifier ion.

BMAA was identified based on retention time compared to D_3BMAA in the same sample, and by the ratios between quantifier and qualifiers which had to be within a 20% relative range of the same ratios in the calibration standards. DAB and AEG were not quantified in this study, but only included in the analysis to ensure that there was no co-elution with BMAA (Figure S1.1.). BMAA was quantified against an external calibration curve and each sample was corrected for D_3BMAA recovery. LOD (based on signal to noise (S/N) ratio for all three transitions of at least 3 in a calibration standard) for BMAA was an injected amount of 34 fmol (2 µg/L), LOQ (S/N ratio of the quantifier at least 10, S/N ratio of the two qualifiers at least 3) was 84 fmol (5 µg/L). Response was linear ($R^2 = 0.995$) within the concentration range of 5 µg/L to 100 µg/L.

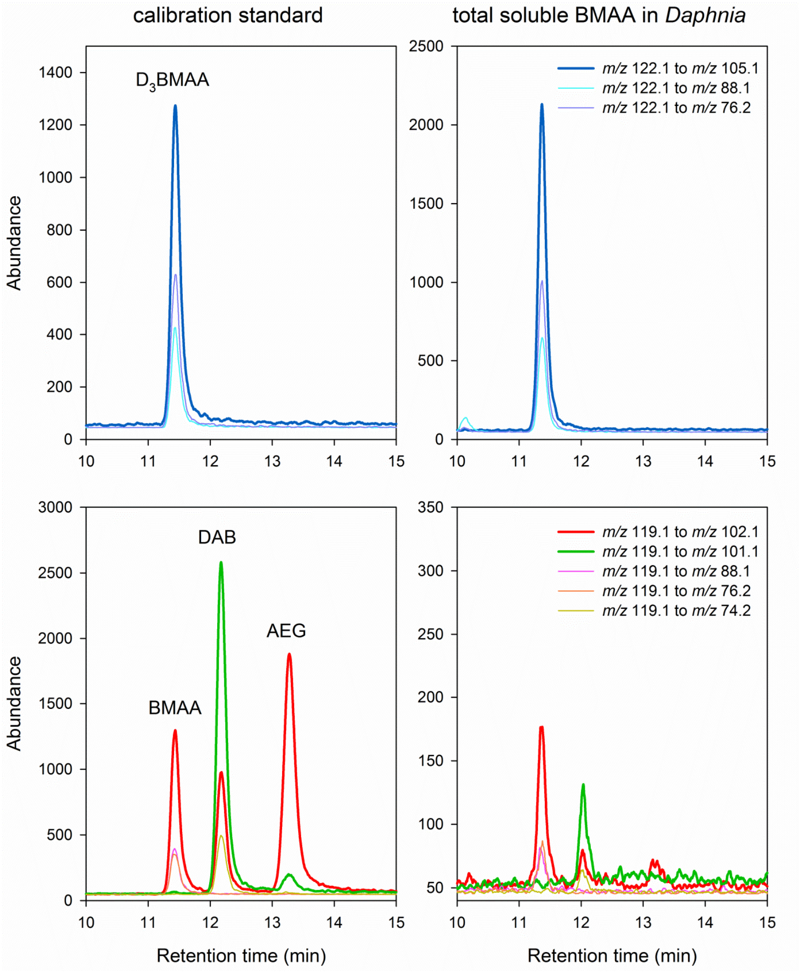

Figure S1.1. Chromatograms of underivatized LC-MS/MS analysis of a calibration standard (left panels) and the total soluble BMAA fraction of a *Daphnia* sample (right panels). Quantifiers are indicated by bold lines, qualifiers are indicated by normal lines. The calibration standard contains 50 µg/L of D_3BMAA, BMAA, DAB and AEG.

S2: Methods and Results of the Derivatised Protocol (Protocol D)

S.2.1. Main Steps Workup

The protocol used for derivatized LC-MS/MS analysis was adapted from a previously published and validated method [6]. This protocol was used on all sample types for the determination of total BMAA. The samples were extracted with aqueous methanol, hydrolysed, cleaned up by chloroform extraction and Isolute HCX-3 SPE, derivatised and concentrated before LC-MS/MS analysis (Figure S2.1).

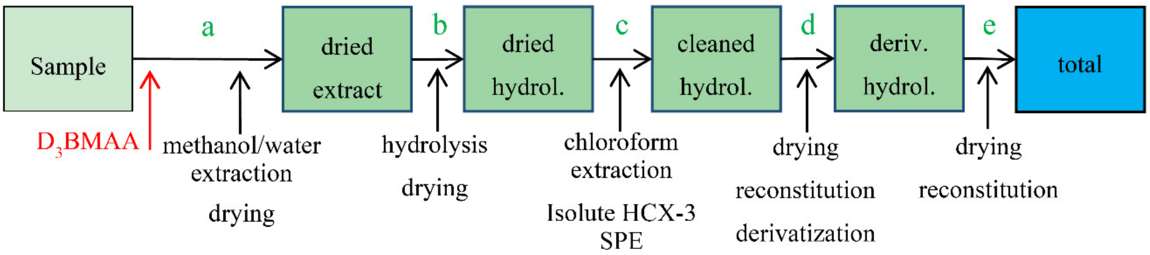

Figure S2.1. Sample preparation scheme for the analysis of derivatized BMAA (protocol D). The main steps are indicated by letters **a** to **e**.

S.2.2. Detailed Protocol

Total BMAA was determined in 0.5 mg of cycad seed, *Daphnia* and seafood sample, 2 mg of cyanobacterial and brain samples was used. Directly after weighing, 80 ng of L-BMAA was added to half of the brain samples. To all samples, 6 µL of a 100 µg/L D_3BMAA solution was added. Samples were subsequently extracted for 10 min in 500 µL 80% methanol in water (*v*/*v*) in an ultrasonic bath at room temperature. The samples were then dried down in a speedvac, and transferred to glass vials using 150 µL of 6 M HCl. The vials were closed and the samples were hydrolyzed overnight in an oven at 110 °C. After hydrolysis, 300 µL of water was added to the vials, and the samples were filtered for 5 min at 16000× *g* over a 0.2 µm cellulose acetate filter. After drying in a speedvac, the samples were reconstituted in 550 µL water, and 1000 µL of chloroform was added. After manually shaking for 5 min, the samples were centrifuged for 3 min at 16000× *g* and 500 µL of the aqueous layer was transferred to a new Eppendorf tube. Water plus 0.2% formic acid (500 µL) was added and the samples were cleaned up by SPE. HCX-3 cartridges (100 mg, Isolute, Uppsala, Sweden) were conditioned with 1 mL of methanol and 1 mL of water with 0.1% formic acid. Samples (1 mL) were loaded, washed with 1 mL of water plus 0.1% formic acid (pH 2.7) and 1 mL of methanol. Samples were eluted by the addition of 2 × 800 µL of NH_4OH in methanol (prepared by adding 2.6% of a 25% NH_4OH solution to 97.4% of methanol (*v*/*v*)). The eluates were dried in a speedvac and reconstituted in 20 µL 20mM HCl. Samples were then derivatized by adding 60 µL of borate buffer and 20 µL of AQC (Waters) derivatization reagent. The derivatized samples were again dried in a speedvac, and reconstituted in 30 µL of a water/acetonitrile solution (95:5 *v*/*v*). Samples were stored at −20 °C before analysis.

As the UPLC method used for the analysis of derivatized samples [6] could not be reproduced on our LC system, we adapted the method for derivatized BMAA analysis as described in [3] to the used LC-MS/MS system to ensure separation of AEG from DAB and BMAA. Chromatography was performed on a Zorbax Eclipse AAA 4.6 × 75 mm, 3.5 µm column (Agilent, Waldbronn, Germany) with the same mobile phases as for the underivatized analysis. The following gradient was applied: 0 min 1% A; 4 min 2% A; 8 min 5% A; 24 min 10% A; 26–30 min 50% A; 30–42 min 1% A with linear increases between the steps. During the first 6 and the last 12 min of each run, the flow was directed to waste. Flow rate was 1 mL/min, injection volume 10 µL and column temperature 40 °C. Source settings were: drying gas temperature 300 °C, drying gas flow 5 L/min, nebulizer pressure 45 psi, sheath gas temperature 400 °C, sheath gas flow 11 L/min, capillary voltage 2500 V, nozzle voltage 500 V. Both quadrupoles were operated in unit mode, and the ESI source was operated in positive mode.

MS/MS settings and transitions monitored in MRM for D3BMAA, BMAA, DAB and AEG are shown in Table S2.1.

Table S2.1. MS/MS settings and MRM transitions for derivatized analysis.

Compound	Precursor	F [1]	Quant [2]	CE [3]	Qual [4]	CE	Ratio [5]	Qual	CE	Ratio
	m/z	V	*m/z*	V	*m/z*	V	%	*m/z*	V	%
D3BMAA	462	134	171	35	145	16	12	122	16	27
BMAA	459	143	171	32	258	25	3	119	16	28
DAB	459	134	171	32	315	12	-	-	-	-
AEG	459	134	171	32	214	35	-	-	-	-

[1] Fragmentor voltage, [2] Quantifier ion, [3] Collision energy, [4] Qualifier ion, [5] Ratio between areas of qualifier and quantifier ion.

BMAA was identified based on retention time compared to D3BMAA in the same sample, and by the ratios between quantifier and qualifiers which had to be within a 20% relative range of the same ratios in the calibration standards. DAB and AEG were not quantified in this study, but only included in the analysis to ensure that there was no co-elution with BMAA (Figure S2.2). BMAA was quantified against an external calibration curve and each sample was corrected for D3BMAA recovery. Calibration standards were prepared in 20 mM HCl, derivatized in the same way as the samples, and subsequently dried down and dissolved in water/acetonitrile as described above. For BMAA, LOD and LOQ were similar: an amount of 45 fmol on column (corresponding to a concentration of 1 µg/L before derivatization and concentration). Response was linear (R^2 = 0.995) within the concentration range of 1 µg/L to 100 µg/L.

S.2.3. D3BMAA Recovery

D3BMAA recovery was below 10% in all samples analyzed with protocol D which is consistent with a recent independent evaluation of a similar protocol [7]. The majority of the analyte is lost during SPE: when during method development a D3BMAA solution was subjected to SPE, without matrix or previous extraction, and the eluate was derivatized directly after drying (step c and d in Figure S2.1), recovery was 37% (SD 7.9, n = 3). When this test was repeated with inclusion of the final concentration step (step e in Figure S2.1), no extra losses occurred (recovery 34%, SD 9.0, n=3). In addition to losses during SPE, strong signal suppression has been reported for this method [7]. Because of the low recovery achieved, it was not possible to determine BMAA concentrations in samples using this protocol.

A recovery of 63.3% has been reported for a similar method where the final concentration step was omitted, combined with alternative chromatography and MS/MS detection [8]. However, this value does not cover full recovery, as it is based on the ratio between a spike added before workup and a spike added before derivatization. Losses that occur during and after derivatization (like signal suppression) were therefore not taken into account. As the optimum sample protein to derivatization reagent ratio (0.005 to 0.25 µg protein/µL before derivatization [9]) is greatly exceeded in this method, it is necessary to determine the efficiency of derivatization.

The D3BMAA recovery of the protocol used in our study was low, but this is not inherent to AQC based LC-MS/MS methods as in our case, a substantial part of the D3BMAA got lost during workup. If coupled to appropriate workup protocols, AQC based methods can give good recoveries, values ranging from 61% to 99% have been reported for AQC derivatized LC-MS/MS analysis of BMAA (e.g., [3,10,11]).

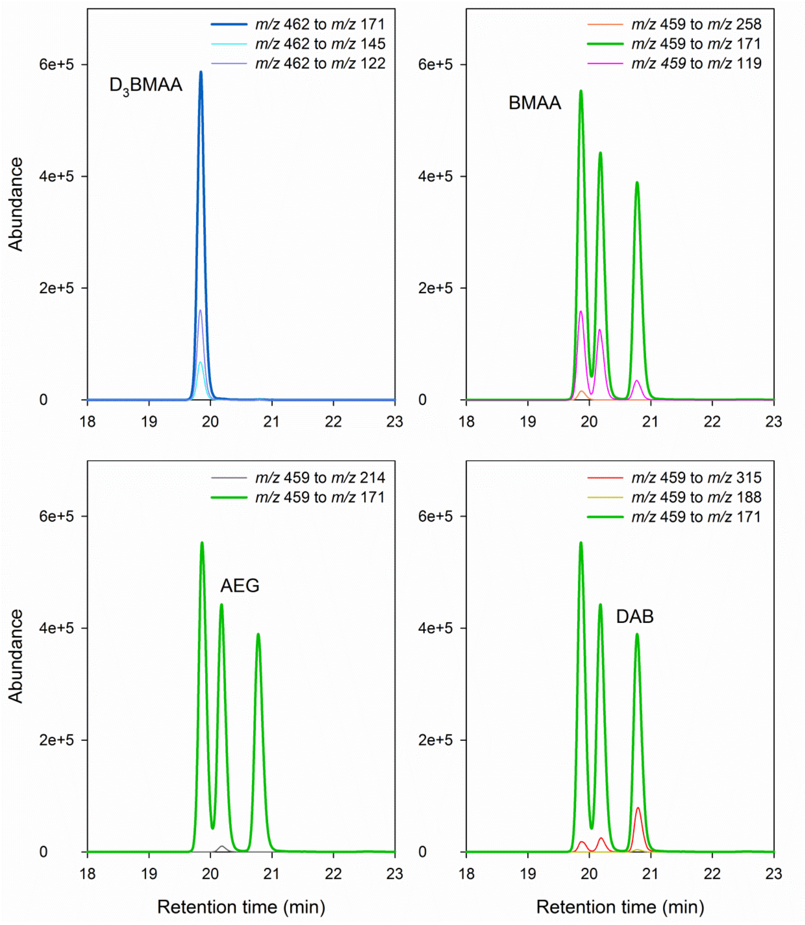

Figure S2.2. Chromatogram of derivatized LC-MS/MS analysis of a calibration standard containing 500 µg/L of D3BMAA, BMAA, DAB and AEG. Quantifiers for D3BMAA and BMAA are indicated by bold lines, qualifiers are indicated by normal lines.

S3: Supplementary Figure

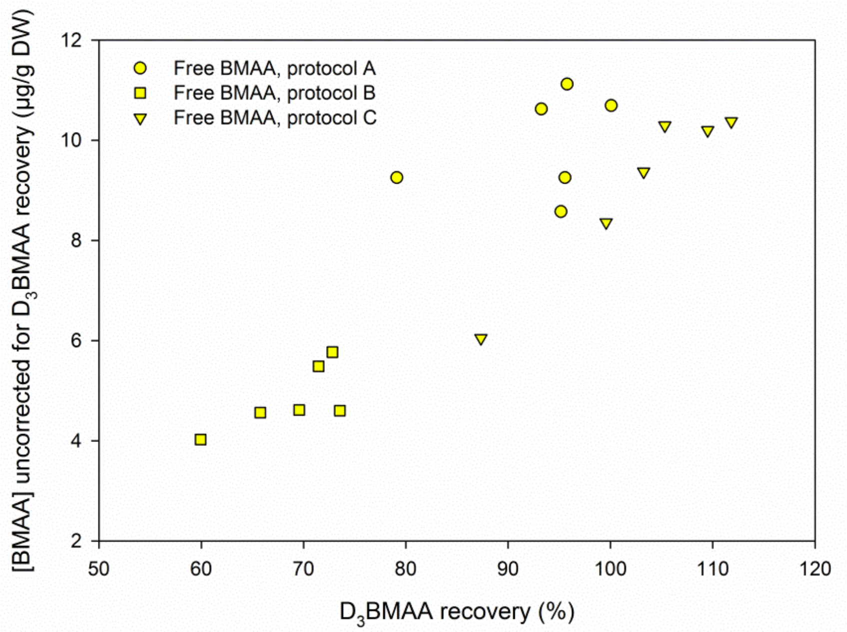

Figure S3. *Cont.*

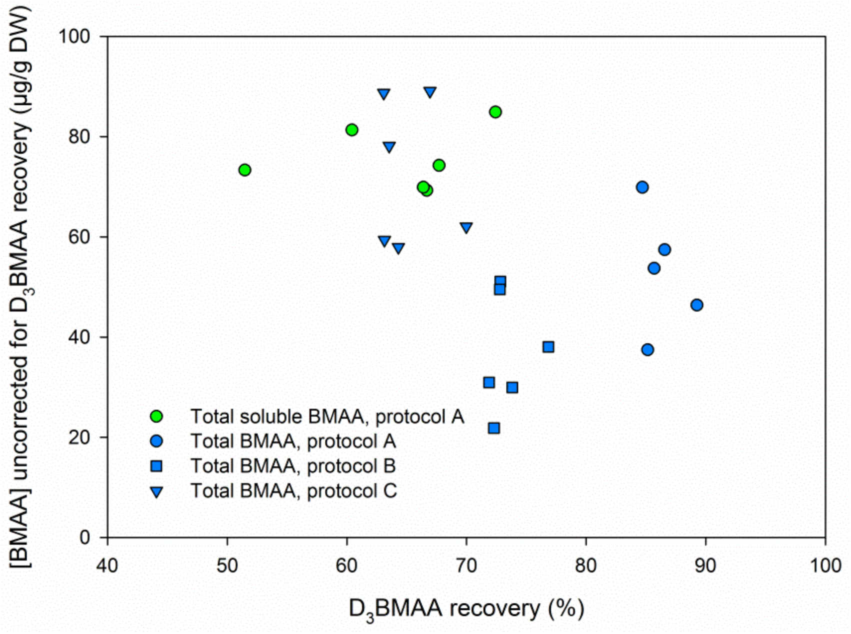

Figure S3. Relation between D3BMAA recovery and the BMAA concentration determined in cycad seeds by underivatized LC-MS/MS analysis, uncorrected for D3BMAA recovery. The upper panel shows free BMAA, the lower panel shows total soluble BMAA and total BMAA.

References

1. Tollrian, R. Neckteeth formation in *Daphnia pulex* as an example of continuous phenotypic plasticity: Morphological effects of *Chaoborus kairomone* concentration and their quantification. *J. Plankton Res.* **1993**, *15*, 1309–1318.
2. Andersen, R.A. *Algal Culturing Techniques*; Elsevier Academic Press: Burlington, MA, USA, 2005.
3. Faassen, E.J.; Gillissen, F.; Lürling, M. A comparative study on three analytical methods for the determination of the neurotoxin BMAA in cyanobacteria. *PLoS ONE* **2012**, *7*, e36667, doi:10.1371/journal.pone.0036667.
4. Combes, A.; El Abdellaoui, S.; Vial, J.; Lagrange, E.; Pichon, V. Development of an analytical procedure for quantifying the underivatized neurotoxin β-*N*-methylamino-L-alanine in brain tissues. *Anal. Bioanal. Chem.* **2014**, *406*, 4627–4636.
5. Faassen, E.J.; García-Altares, M.; Mendes e Mello, M.; Lürling, M. Trans generational effects of the neurotoxin BMAA on the aquatic grazer *Daphnia magna*. *Aquat. Toxicol.* **2015**, *168*, 98–107.
6. Jiang, L.; Kiselova, N.; Rosén, J.; Ilag, L.L. Quantification of neurotoxin BMAA (β-*N*-methylamino-L-alanine) in seafood from Swedish markets. *Sci. Rep.* **2014**, *4*, 6931, doi:10.1038/srep06931.
7. Lage, S.; Burian, A.; Rasmussen, U.; Costa, P.R.; Annadotter, H.; Godhe, A.; Rydberg, S. BMAA extraction of cyanobacteria samples: Which method to choose? *Environ. Sci. Pollut. Res.* **2015**, in press.
8. Jiang, L.; Johnston, E.; Åberg, K.M.; Nilsson, U.; Ilag, L.L. Strategy for quantifying trace levels of BMAA in cyanobacteria by LC/MS/MS. *Anal. Bioanal. Chem.* **2012**, *405*, 1283–1292.
9. Waters. *Waters AccQ-Tag Chemistry Package Instruction Manual*; Waters Cooperation: Milford, DE, USA, 1993.
10. Christensen, S.J.; Hemscheidt, T.K.; Trapido-Rosenthal, H.; Laws, E.A.; Bidigare, R.R. Detection and quantification of β-methylamino-L-alanine in aquatic invertebrates. *Limnol. Oceanogr. Methods* **2012**, *10*, 891–898.
11. Jiao, Y.; Chen, Q.; Chen, X.; Wang, X.; Liao, X.; Jiang, L.; Wu, J.; Yang, L. Occurrence and transfer of a cyanobacterial neurotoxin β-methylamino-L-alanine within the aquatic food webs of Gonghu Bay (Lake Taihu, China) to evaluate the potential human health risk. *Sci. Total Environ.* **2014**, *468–469*, 457–463.

© 2016 by the authors; licensee MDPI, Basel, Switzerland. This article is an open access article distributed under the terms and conditions of the Creative Commons by Attribution (CC-BY) license (http://creativecommons.org/licenses/by/4.0/).
